# Scaling in Colloidal and Biological Networks

**DOI:** 10.3390/e22060622

**Published:** 2020-06-04

**Authors:** Michael Nosonovsky, Prosun Roy

**Affiliations:** Department of Mechanical Engineering, University of Wisconsin—Milwaukee, 3200 North Cramer St., Milwaukee, WI 53211, USA; prosun@uwm.edu

**Keywords:** allometry, droplet clusters, colloidal crystals, biomimetics, network topology

## Abstract

Scaling and dimensional analysis is applied to networks that describe various physical systems. Some of these networks possess fractal, scale-free, and small-world properties. The amount of information contained in a network is found by calculating its Shannon entropy. First, we consider networks arising from granular and colloidal systems (small colloidal and droplet clusters) due to pairwise interaction between the particles. Many networks found in colloidal science possess self-organizing properties due to the effect of percolation and/or self-organized criticality. Then, we discuss the allometric laws in branching vascular networks, artificial neural networks, cortical neural networks, as well as immune networks, which serve as a source of inspiration for both surface engineering and information technology. Scaling relationships in complex networks of neurons, which are organized in the neocortex in a hierarchical manner, suggest that the characteristic time constant is independent of brain size when interspecies comparison is conducted. The information content, scaling, dimensional, and topological properties of these networks are discussed.

## 1. Introduction

Scaling methods and dimensional analysis are widely used in various areas of physics. The concepts of fractals (scale-free objects), power exponents, and near-critical behavior are central to the study of scaling. One particularly interesting field of application is biophysical problems, where both experimental observations and theoretical explanations have been suggested of how various quantitative characteristics of a living organism (for example, the rate of metabolism) depend on its mass and linear size. The area is often referred to as allometry [1,2,3,4,5,6].

One of the most widely known examples of an allometric scaling relationship is the empirical Kleiber law, which is based on a comparison of the metabolism rates, *B*, of different species. Kleiber [1] established a power law dependency with the exponent ¾ of *B* upon the mass of an animal, B∝M^3/4^. The explanation of the value of the exponent ¾ was suggested in an influential paper by West et al. [2], who used a fractal model of branching of blood vessels serving a certain volume with the conservation of the cross-sectional area of the vessels and of the volume covered at every stage of branching. Despite its shortcomings [3,4,5,6], the fractal theory by West et al. [2] remains the main explanation of the allometric scaling exponents, and it can likely be expanded on such topics as the analysis of the ergodicity in the vascular network [7].

Besides the biophysical applications, scaling problems are also prominent in those areas of physics, where the so-called intermediate asymptotic behavior is found [8]. A typical example of an intermediate asymptotic process would be a transition from a single object to a thermodynamic description of a continuum medium. The intermediate ensemble of a few natural or artificial objects, which bridges between a single particle and continuum matter, is often called a cluster, and it possesses many properties absent from both single-particle and continuum medium description [9]. This includes scaling properties of large clusters and their description by means of the clustering coefficients, as it will be discussed later.

In the last 20–30 years, partially due to the success of computer networks such as the internet, the concept of networks, which originates in the mathematical theory of graphs, became a particularly successful and popular method of modeling various processes and systems [9,10,11,12]. Network methods can be applied in such diverse areas as the materials science, where they could represent, for example, a force network and the packing of granular material, or as the colloidal science, where they represent colloidal and droplet crystals. Network description also emerges naturally in the study of biological systems such as the vascular, pulmonic, or neural systems, including the brain. Networks as mathematical objects possess certain topological and, in particular, scaling properties including those similar to the properties studied by allometric approaches. Fractal properties are often related to self-organizing phenomena such as self-organized criticality (SOC)—a particular type of self-organization, when a dynamical system tunes itself to a critical state. SOC plays a role in several areas from wetting and avalanches in granular material to the development and functioning of the brain.

Besides the scaling properties of networks, their information-related properties are important. This is because many networks are used for information processing. The amount of information contained by a network can be estimated by calculating its entropy, such as the Shannon entropy. This universal property is applicable to different types of networks from clusters of colloidal particles to cortical networks of neurons in the human brain.

Network study is a highly interdisciplinary area. In this paper, we analyze and review the application of networks to several different areas, namely, to the colloidal and surface science and to the biological systems including the vascular, neural, and immune networks. While these areas belong to different domains of science, the application of networks shows a significant similarity of analytical methods. Both areas involve scaling and power exponents, as well as scale-free behavior. We discuss approaches to establishing scaling laws in these systems, investigating their dimensional and topological properties and using entropic methods to estimate information content.

## 2. Scaling Concepts in Networks and Clusters

Mathematically, networks are graphs representing either symmetric or asymmetric relations between discrete objects. Although the topology and scaling behavior of various networks has been studied since the 1930s [13], their especially extensive study started in the 1990s. A number of important properties have been discovered experimentally in real-life networks including the scale-free and small-word network behavior.

### 2.1. Scale-Free and Small-World Networks

The number of connections of a given node in a network with other nodes is called the *degree* of the node, *k*. The probability distribution function of the degree of the node in the entire network is called the *degree distribution*, *P*(*k*). An important class of large networks has the degree distribution functions, which approximately follow a power law

(1) $$P\left(k\right)\propto k^{-a}.\;$$

where *a* is a constant, usually in the range 2 < *a* ≤ 3. Such a network is called a *scale-free* network. For *a* ≤ 3, the standard deviation of the degree diverges (in particular, there is a logarithmic divergence for *a* = 3), and the network lacks a characteristic degree; hence, the name a “scale-free network”.

Scale-free networks are abundant in nature and culture including various biological, social, and technical systems, such diverse as citation and co-author scientific networks, the internet, and protein–protein interaction [10]. The commonly used algorithm to create a scale-free network is the Barabási and Albert model (BA) model [11]. The BA model is often compared with the random graph Erdős–Rényi (ER) model [12] of a random network. Unlike a scale-free network, the random ER network has the average degree value, *k_ave_*.

Scale-free behavior is common not only in networks. It emerges in various areas of physics and it is associated with the fractal phenomena and with the near-critical behavior close to the phase transition. Many scale-free systems are governed by so-called *self-organized criticality* (SOC), whose characteristic signature is the one-over-frequency noise and avalanche-like behavior. Self-organized criticality is found in systems where energy is accumulated and then propagated in an avalanche-like manner including wetting [14] and friction [15].

Scale-free networks have some nodes with a degree that greatly exceeds the average, and such highly connected nodes are called *hubs*. In real-life networks, hubs often have specificity; for example, they can connect parts of a network; in social networks, hubs may correspond to “celebrities”, etc. Smaller hubs are present next to major hubs, thus creating a hierarchical organizational structure of a scale-free network. Scale-free networks have high resilience or tolerance toward failure. If a number of nodes, including hubs, are removed from the network, it still remains connected.

Quantitatively, the resilience is characterized by the critical percolation threshold, *p_c_*, or the fraction of nodes, which should be kept to sustain the connectivity [16]. Thus, for an infinite network with 2 < *a* < 3, the critical percolation is zero, *p_c_* = 0, which means that removing randomly any fraction of the network cannot destroy it. For a finite scale-free network with *N* nodes, $p_{c}\sim N^{-1/3}$; thus, a large network such as the internet with $N>10^{9}$ will not be destroyed until more than 99.9% of nodes are removed in a random manner (however, it can be destroyed if high-degree nodes are removed preferentially). Note that, as opposed to the scale-free network, in an ER random network, the threshold percolation is non-zero, and it is inversely proportional to the average degree $p_{c}\sim 1/k_{ave}$. This consideration may be applicable to the spreading of infectious diseases, such as Covid-19, when emergency closure measures of a sufficient number of public institutions can prevent disease spreading.

A path or a walk within a network is a sequence of incident nodes and edges. Nodes or edges can appear in the same path more than once. The geodesic path is the shortest path between any two nodes, whereas the geodesic distance between two nodes is the minimum number of edges connecting these two nodes or the shortest path length between them. A network is considered a *small world* if a typical geodesic distance between two randomly chosen nodes, *L*, is proportional to the logarithm of the total number of nodes, *N*, in the network

(2) L∝log(N).

**Figure 1:**
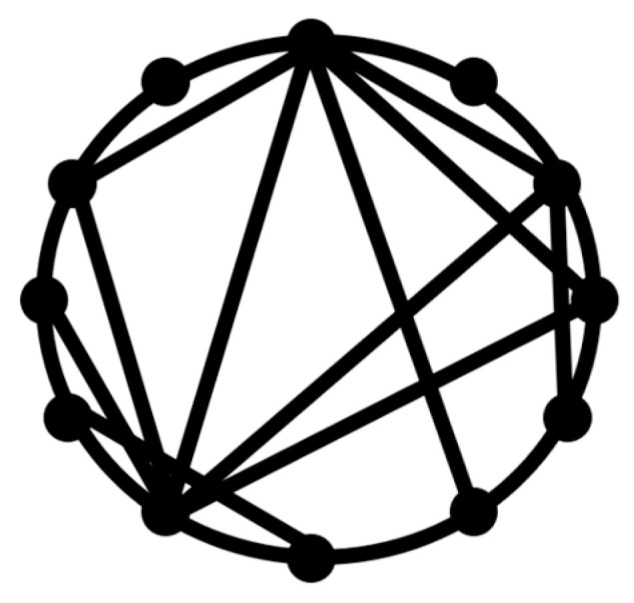
Small-world network concept. While some nodes have connections only with their neighbors, edges connecting with remote nodes provide short geodesic paths.

The small-world concept implies that despite their large size, in most networks, there is a relatively short path between any two nodes (Figure 1). The commonly studied type of the small-world networks is the Watts and Strogatz [17] model.

Clusters are defined as collections of somewhat similar, but not necessarily identical objects. In physics and chemistry, clusters are often built of liquid droplets or small solid particles, such as nanoparticles, and colloidal particles [9]. From the physical point of view, they occupy an intermediate position between individual objects and bulk material consisting of a large number of objects, to which the methods of statistical physics and thermodynamics are often applied. Since clusters bridge between individual particles and bulk matter, they may have certain unique collective properties absent in both individual objects and in bulk materials.

Given that there are interactions between particles in a cluster, the latter can be modeled by a network. A network is a graph, which consists of nodes (vertices) connected by edges. The clustering coefficient is defined as a ratio of the number of closed triplets to all triplets. This coefficient is a measure of the degree to which nodes in a graph tend to cluster together. It is used to quantify aggregation in granular media, indicating the degree of clustering of particles in the cluster.

### 2.2. Self-Organized Criticality and Percolation

Two physical concepts relevant to the self-organization of various networks are self-organized criticality (SOC) and percolation [14,15,16,18,19]. There is a big class of dynamical systems that operate in such a manner that they always tune themselves to the critical state, where the stability of the system is lost. Since the 1980s, it has been suggested that a very specific type of self-organization, called SOC, plays a role in diverse “avalanche-like” processes. Typically, energy is accumulated in these systems until the critical state is reached, and then energy is suddenly released. Examples are various avalanche systems, including those describing landslides, earthquakes, and frictional stick-slip. A random perturbation can trigger an avalanche (or a slip event) in such a system. The magnitude of the avalanche cannot be predicted in advance, because it is random. After the release, the system returns to the stable state for some time until the next event is triggered. The amplitudes of the events have statistical characteristics of critical behavior, such as universality, critical exponents, and fractality.

A famous example is the power law, which relates the frequency and the magnitude of earthquakes, known as the Gutenberg–Richter law. For example, similar behavior is observed in frictional stick-slip systems and in many other systems [15].

**Figure 2:**
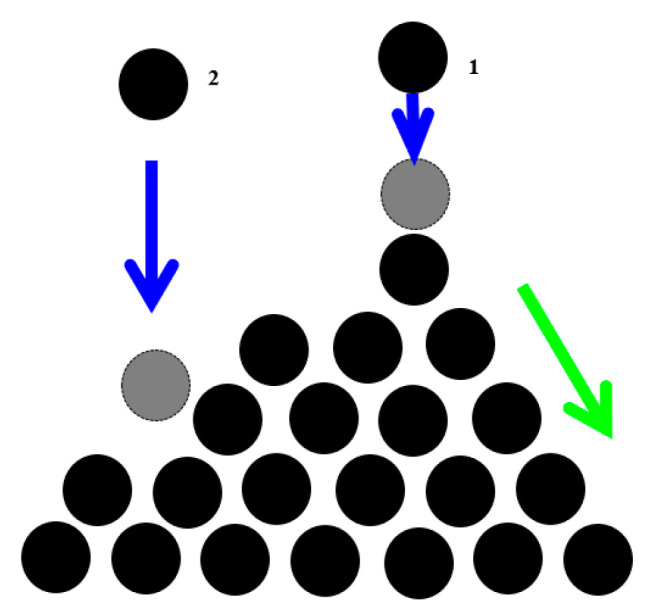
The sand-pile conceptual model of self-organized criticality (SOC). The pile tends to have a slope angle defined by the friction between grains. Adding one new grain to the pile may have no effect (grain is at rest) or it may cause an avalanche. The magnitude and frequency of avalanches are inversely related (based on [15]).

The best-studied example of SOC is the “sandpile model”, which represent a conical pile of sand with new grains of sand randomly placed into the pile (Figure 2). When the slope exceeds a threshold value (the critical slope angle is related to the coefficient of dry friction between the grains), a grain would move down the slope. Placing a random grain at a particular site may have no effect, or it may trigger an avalanche that will affect many sites at the lattice. Thus, the response does not depend on the details of the perturbation. It is worth mentioning that the scale of the avalanche may be much greater than the scale of the initial perturbation.

The concept has been applied to such diverse fields as physics, cellular automata theory, biology, economics, sociology, linguistics, and others. There are typical external signs of an SOC system, such as the power-law behavior (the magnitude distribution of the avalanches) and the ‘one-over-frequency’ noise distribution (the amplitude of random fluctuations is inversely proportional to the frequency). Of course, not every system with a one-over-frequency spectrum is a SOC system. The one-over-frequency noise (referred to also as the white noise) may be present also in non-SOC systems.

**Figure 3:**
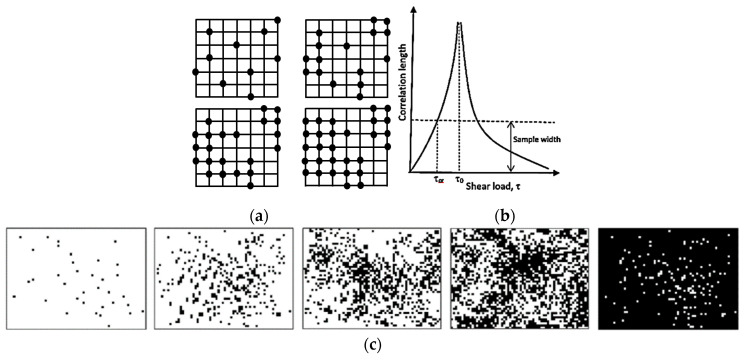
Percolation. (**a**) A 2D torus model with a two active neighbors local update rule (if there are two active neighbors, the edge becomes active), showing that the first four iteration steps at the eighth step all sites become active [18]. (**b**) A typical dependency of the correlation length on the shear load for an avalanche. At the critical value of the load, τ_0_, the correlation length approaches the infinity. (**c**) With the increasing normal load, the size of slip zone spots (black) increases. A transition to the global sliding is expected when the correlation length approaches infinity.

Another important concept, which is related to SOC, is the percolation [15,18]. Typically, during percolation, a certain controlled parameter is slowly changed; for example, nodes are removed from a network or conducting sites are added (Figure 3a), or shear force is increased in a system with friction. When a critical value of the controlled parameter is achieved, the avalanche can be triggered and a corresponding output parameter, such as the correlation length, may reach infinity (Figure 3b). For example, the correlation length can characterize the average size of black or white islands on a field of the opposite color, when random pixels are added (Figure 3c). At the critical point, the configuration is fractal (an infinite set of white islands on the black background forming larger black islands on the white background, and so on).

## 3. Networks in Colloidal Science: Granular Material, Colloidal Crystals, and Droplet Clusters

The topological concepts from the theory of networks turn out to be useful for physical characterization of packing of granular material, colloidal crystals, and clusters of droplets and colloidal particles.

### 3.1. Granular Material

**Figure 4:**
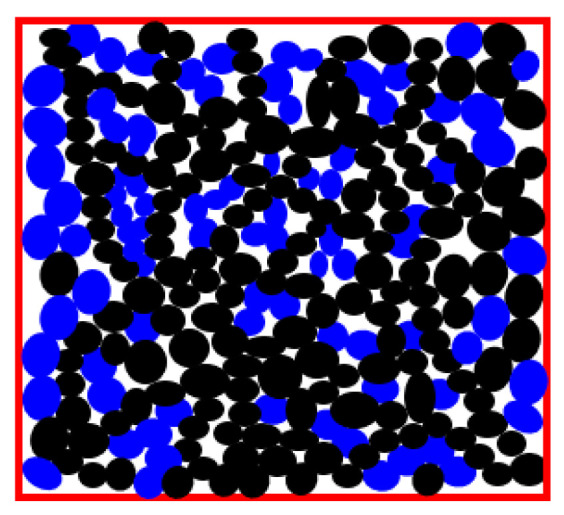
Model of jamming in a granular media as percolation of a force network (the concept based on [20,21]). The force is transmitted through chains of connecting grains shown in black.

The so-called *force network* is important for the understanding of packing of granular material. Figure 4 shows a granular material (blue and black circles represent granules) flowing in a channel. Some grains are tightly packed and apply a force upon their neighbors, while others are loose and do not transmit force. With increasing pressure in the channel, the number of force-transmitting grains (black) increases, and when the chain of black grains through the entire width of the channel, it is jammed, which is called the jamming transition.

The force network connects centers of mass of each pair of grains that have a force transmitting contact. Such network presentation provides key insights for understanding the mechanical response of a soil or sand heap. Moreover, percolation, i.e., the formation of a force-transmitting chain in such a network, corresponds to the jamming transition in the granular material [20,21]. The percolation phenomenon in application to networks will be discussed more in detail in the consequent section. Near-percolation behavior is known to demonstrate scale-free features.

**Figure 5:**
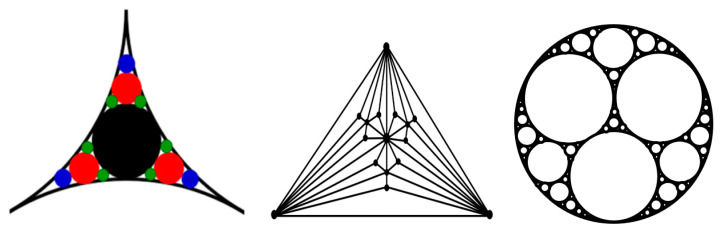
Apollonian packing and a corresponding graph (the concept based on [22]).

In particular, the small-world effect and scale-free behavior were reported for packing problems related to aggregation of granular media, which employs the so-called “Apollonian packing” [22]. For the simple packing of identical (monodisperse) particles, the force networks do not possess self-similar, scale-free, or small-world properties. However, to achieve high packing densities, the Apollonian construction can be employed (Figure 5). Such construction involves a multiscale set of circles with smaller circles fitting the space between larger ones. This may be needed for high-performance concrete (HPC) and certain ultra-strong ceramics. Andrade et al. [22] found that the force networks resulting from the Apollonian construction, which they called Apollonian networks (ANs), have many special properties, including the scale-free and small-world effects.

### 3.2. Droplet Clusters

**Figure 6:**
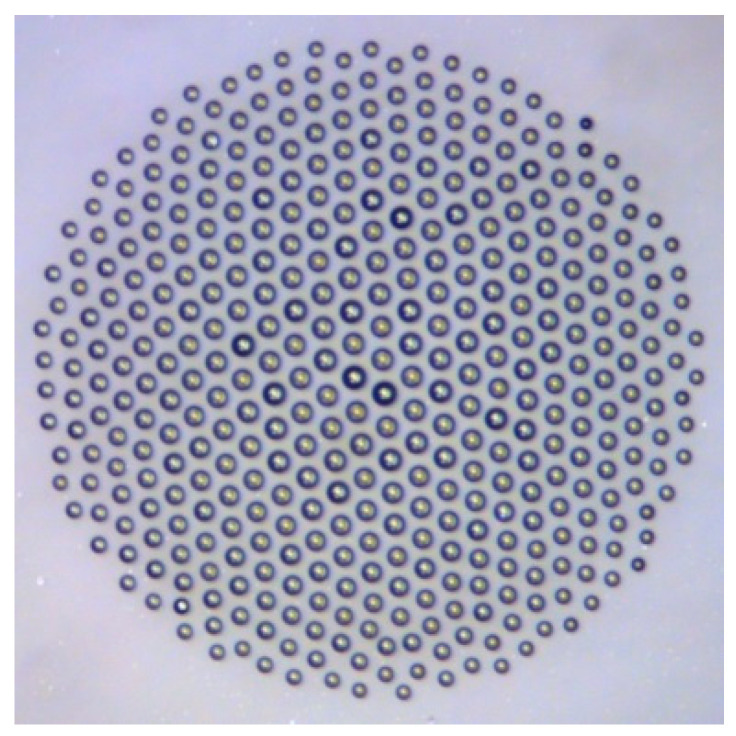
A hexagonally ordered 2D droplet cluster levitating over a water surface. The size of the frame is 0.75 mm (credit: Dr. A. Fedorets, based on [26]).

Even more interesting phenomena where the theory of networks can be applied are small droplet clusters [23,24,25,26,27] and small colloidal crystals. Self-assembled clusters of condensed microdroplets (with the typical diameter of dozens of microns) are formed in an ascending flow of vapor and air above a locally heated thin (approximately 1 mm) layer of water. The droplets form a 2D monolayer levitating at a low height (comparable with their radii) where their weight is equilibrated by the drag force from the ascending vapor flow. Due to an aerodynamic interaction (repulsion) between the droplets and their migration toward the center of the heated spot, they tend to form an ordered structure (Figure 6).

For large clusters consisting of many dozens or hundreds of droplets, a hexagonally symmetric (honeycomb) structure is typically formed. However, for small clusters, more complex symmetric structures can form in comparison with those in large clusters. For example, the applicability of the mathematical *ADE*-classification or the so-called simply laced Dynkin diagrams has been suggested [27]. Small clusters can be used for the in situ tracking of bioaerosols and biomolecules [25].

The method of Voronoi entropy is applied to characterize quantitatively the degree of orderliness of the geometric arrangement of the droplet clusters. The Voronoi entropy is calculated using the so-called Voronoi tessellation, when an image is divided into a set of polygons. Each polygon consists of all points closer to the center of a particular droplet than to any other droplet. The Voronoi entropy is then calculated as

(3) $$S_{vor}=-\sum_{n}P_{n}\ln P_{n},$$

where *P_n_* is the fraction of polygons with *n* sides or edges [26].

In general, three scaling laws relevant to the Voronoi entropy in such colloidal systems are Lewis’ law, the Desch law, and the Aboav law. Lewis observed a linear relationship between the average area of a typical *n*-gon and *n* for various random 2D cellular mosaics. The Desch law states a linear relationship between the perimeter of polygons and the number of their edges, while the Aboav law relates the average number sides of a Voronoi cell neighboring an *n*-gon a+b/n, where *a* and *b* are constants [26].

### 3.3. Colloidal Crystals

**Figure 7:**
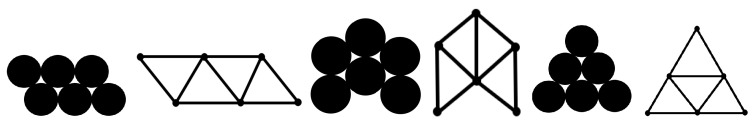
Schematic of colloidal particles forming small clusters (concept based on [28]).

Unlike liquid water droplets, colloidal particles are solid, and they can form small clusters, which can levitate due to acoustic waves or due to another mechanism, but they form a close packed structure. Thus, Perry et al. [28] studied the structural rearrangement in a 2D levitating cluster of solid *sulfate polystyrene* spherical particles. Individual particles in a particular configuration (an excited state) of the cluster may have bonds between them. For a system of six particles, Perry et al. [28] identified a number of seven-bond and eight-bond configurations. In similar systems, Lim et al. [29] reported the transitions from sticky to ergodic configurations in six-particle and seven-particle systems of hard 1.3 μm diameter spheres (Figure 7). The six-particle system can form various arrangements, with different probabilities of these arrangements.

For many distributions of small elements (for example, the words in a language or letters in a text), an empirical power law, such as the Zipf law, is found. The Zipf law is given by the formula

(4) $$P\left(k\right)=\frac{k^{-a}}{\sum_{n=1}^{N}n^{-a}}$$

where *P(k)* is the frequency of an element of rank *k*, *a* is a power exponent, and the denominator is needed for normalization. A power law distribution may be expected for the probabilities of various excited states of the cluster forming a set of rearrangement configurations. It is instructive to investigate the probabilistic distributions and information content in graphs corresponding to small clusters.

**Figure 8:**
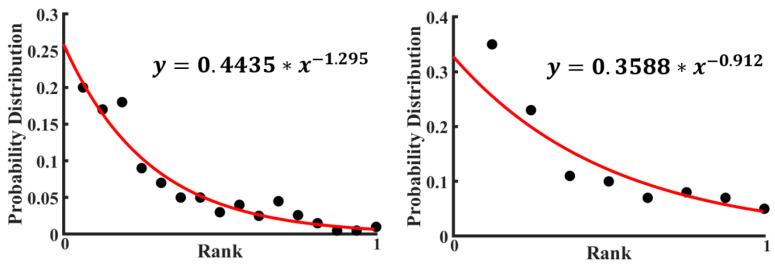
Experimental probability distributions of 8-bond (**right part**) and 7-bond (**left part**) structures. Each point is representing each distinguished structure of a colloidal cluster (based on data from [28]).

For the data by Perry et al. [28], the probability distribution of various configurations was plotted in Figure 8 (the rank is a number of a given configuration). A calculation based on the empirical formula of the Zipf Law (Equation (4)) using the experimental data points has been done to fit those experimental points. Then, a fitted curve has also been plotted in Figure 8 with the data presented in Table 1. The value of the power exponent in the curve fitting equation is almost one, which indicates that the fitted curve is hyperbolic. See also a discussion of 2D colloidal clusters by Janai et al. [30].

The information content of a distribution is characterized by the Shannon entropy, which is given by

(5) $$S=-\sum_{n=1}^{N}p_{n}\log_{2}\left(p_{n}\right).\;$$

where *p_n_* is the statistical probability of the *n*-th state and *N* is the total number of states. The Shannon entropy is used in materials science, for example, as a surface roughness parameter characterizing informational content in the surface given by its profile [15]. The Shannon-entropy-based informational approach is also used for various other aspects of surface science, such as wetting transitions [31] and stick-slip transition [32].

Using the data from Figure 8 and Table 1 and Table 2, the following values were obtained. For the seven-bond configurations, the value of the Shannon entropy *S* = 2.745 was obtained, while for the eight-bond configuration, the value of *S* = 3.400 was obtained. The Shannon entropy provides an estimation of the information content in these configurations; in particular, one could expect that the seven-bond cluster is more random than the eight-bond cluster.

To conclude this section, methods of network science can be used for the analysis of various systems studied by physical chemistry and materials science. These include granular materials, colloidal crystals, and clusters made of small particles or droplets. Many such systems form sets of configurations somewhat similar to the set of symbols (e.g., letters) and characterized by power-law statistical distributions typical for the latter. The power law distribution is also characteristic for scale-free networks, which will be discussed more in detail in the following chapter. The information content of these structures can be estimated using the Shannon entropy approach.

We conclude that using the network representation for colloidal systems, such as the granular material, colloidal crystals made of small rigid particles, and levitating droplet clusters, results in the scale-free behavior and in a number of important scaling relationships such as the Zipf, Lewis, Desch, and Aboav scaling laws.

## 4. Artificial Neural Networks in Surface Science

Another area closely related to colloidal science is surface science. Surface scientists and engineers often deal with parameters of surfaces such as surface roughness, surface free energy, and water contact angle. Usually, these parameters are determined in an experimental manner, and they cannot be predicted from the first physical principles.

A more applied branch of surface science, which deals with friction, roughness, lubrication, and adhesion is called tribology. Tribology deals with such characteristics of contacting surfaces as the coefficient of friction and the wear rate. However, one of the challenges in the tribological studies is that while there is a big amount of experimental data about the frictional, wear, and surface properties of various materials, systems, and engineering components, this interdisciplinary area is highly empirical. Tribology remains a data-driven inductive science.

It has been recently suggested to apply machine learning techniques in order to predict surface wetting properties. Kordijazi et. al. [33] applied a multilayer perception neural network model to study the wetting properties of a ductile iron–graphite composite including complex dependencies between the contact angle, composition, surface roughness, and the time of exposure to liquid. Understanding these correlations allows predicting water-repellent properties of metallic composite materials for the optimized design of novel hydrophobic and superhydrophobic materials.

Artificial Neural Networks (ANNs) are computer models somewhat resembling neural networks in the human brain. ANNs incorporate a series of functions to process the input data and convert them over several stages into the desired output. Since ANN models learn by examples and training, they are suited for storing and retrieving acquired knowledge. A typical ANN model has interconnected nodes that model complex neurons and synapses in the human brain. The knowledge acquired during the training process is stored in the synaptic weights of the inter-nodal connections leading to the ability to represent complex input–output relationships. ANNs learn by examining individual records, generating the prediction for each record, comparing the result with the prediction, and making adjustments. Training makes the network more accurate in predicting new outcomes.

**Figure 9:**
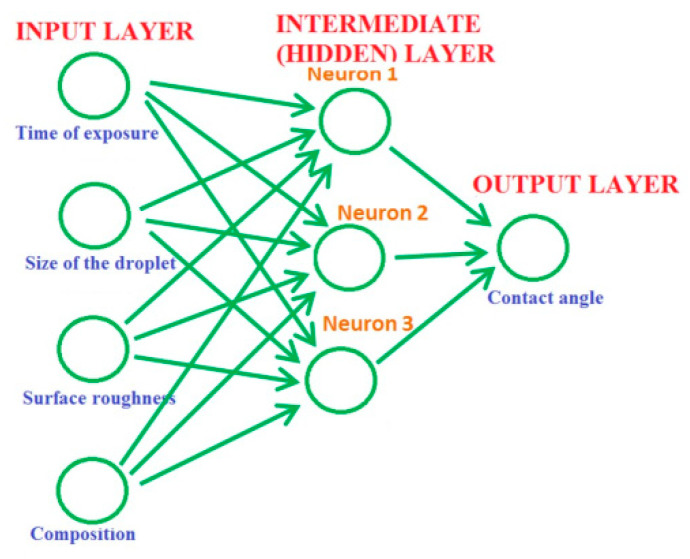
The architecture of Artificial Neural Networks (ANNs) used for the determination of the contact angle (based on [33]).

Typically, a neural network has three parts or layers: the input, intermediate (hidden) layers, and output layer (Figure 9). The input layer with units representing the input data, such as the data about the material composition and surface roughness or conditions of the experiment, for example, the size of the droplets used in wetting experiments, and the time of exposure. One or more hidden layers connect the units with varying connection weights until the results are finally delivered to the units in the output layer. The units in the hidden layer compute their activations based on the data from the input layer and a non-linear transfer function and transmit it to the output layer [33].

ANN models and other machine learning techniques will likely become a popular tool for the analysis of properties of surface and colloidal systems. Note that ANNs themselves do not possess any scaling properties relevant to the study of physical systems. However, ANNs represent a so-called biomimetic approach, because they attempt to mimic learning algorithms in human brains. Therefore, it is of interest to investigate natural neural networks or cortical networks, and these will be discussed in the following section.

## 5. Scaling of Branching Vascular Networks

Let us start the discussion of scaling in biological objects from the scaling rules in branching networks, for which the typical example is the vascular system of mammals. According to the empirical allometric Klieber law [1] formulated in 1932, metabolism rates, *B*, in various species are well approximated by a power-law scaling dependency on the mass of an animal, $B\propto M^{0.75}$. Based on the metabolism rates, one can estimate other parameters in species including the lifespan. The value of the Klieber law exponent, *a* = 0.75, remained mysterious until the seminal paper by West et al. [2] explained it using the fractal model of blood vessel branching. The blood vessels serve a certain volume with the simultaneous conservation of the cross-sectional area of the vessels and of the volume covered at every stage of branching. Despite its shortcomings [3,4,5,6], this theory remains the main explanation of the allometric scaling exponents.

**Figure 10:**
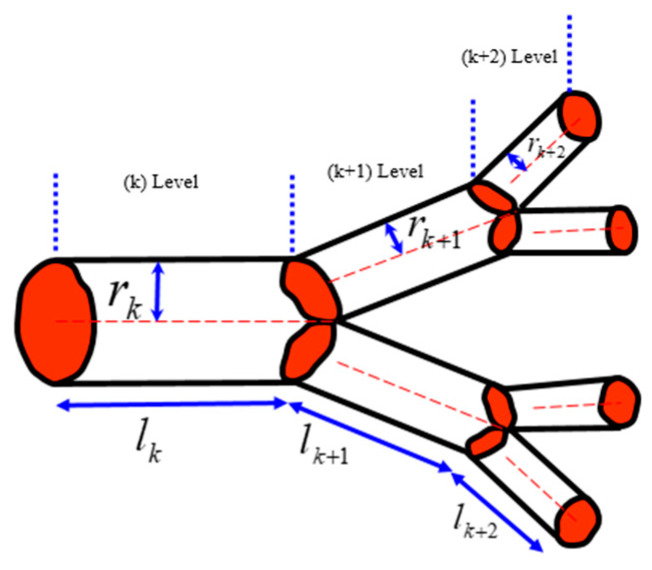
Branching of a vascular network; three levels are shown (based on [7]).

According to West et al. [2], when a tube with the length *l_k_* and radius *r_k_* branches into *n* tubes with the lengths *l_k+1_* = γ*l_k_* and radii *r_k+1_* = β*r_k_* (where γ and β are constants), the volume served by the next-generation tubes and their cross-section area should conserve, which leads to two separate scaling relationships for the constants $\gamma \propto n^{-1/3}$ and $\beta \propto n^{-1/2}$. These relationships are satisfied simultaneously [7]. The area is preserved due to the constant rate of the fluid flow at different hierarchical levels. The volume is preserved, assuming that the same volume in the organism is served by blood vessels of different hierarchical levels (Figure 10).

The total volume of fluid in the vascular system can be calculated as a sum at different levels of the hierarchy using the summation of the geometric series $V=V_{0}\frac{{\left(\gamma \beta^{2}\right)}^{-N}}{1-n\gamma \beta^{2}}$, where *V_0_* is a certain elementary volume (e.g., volume served by a capillary), and *N* is the total number of branch generations. Therefore, the volume scales as $V\propto {\left(\gamma \beta^{2}\right)}^{-N}$. From this, the scaling dependency of the total number of thinnest capillaries as a function of volume is $n^{N}\propto V^{a}\propto {\left(\gamma \beta^{2}\right)}^{-Na}\propto {\left(n^{-4/3}\right)}^{-Na}\propto n^{4Na/3}$ yielding *a* = 3/4, the well-established empirical results known as the Kleiber law, which is based on the assumption of a constant flow rate.

The model by West et al. [2], while simplified, is important at the conceptual level. It was suggested that the fractal scaling of the vascular system may explain the non-ergodicity of the blood flow [7]. Branching in the vascular network provides a classical mechanism for estimating scaling, which can be applied to more complex neuron networks.

## 6. Cortical Neural Networks

In this section, we will review certain aspects of the current knowledge about the cortical networks in human and animal brains related to their scaling and self-organizing properties. This area of neuroscience is rapidly developing and intersects with the network science in many instances. Since this area is less known to biophysicists, colloidal scientists, and engineers, we will introduce some concepts on the structure of cortical networks prior to discussing their scaling and self-organizing properties.

### 6.1. Structure and Properties of Neural Networks of the Human Brain

Let us start from the discussion of the architecture of the cortical network. Neuron connections in the human brain constitute a very complex network of about 10^10^ neurons with more than 10^14^ synapses connecting between them. While it is extremely difficult to study such a complex network, a number of important insights have been achieved, particularly, since the early 2000s. This knowledge was obtained due to novel methods of in vivo observation of neural activity, including the electroencephalography (EEG), functional magnetic resonance imaging (fMRI), diffusion tensor imaging (DTI), two-photon excitation microscopy (TPEF or 2PEF), and positron emission tomography (PET).

Many insights were also achieved using the comparison or analogy of the human brain with the neural system of much simpler organisms, such as the nematode Caenorhabditis elegans (a tiny worm with the size of less than 1 mm), which has only 302 neurons and about 6398 synapses. Since a complete connectome (a map of neuron connections) and genome have been obtained and published for *C. elegans*, in 1986 [34], this worm serves as a model organism for genetic and neurological research including, for example, 3D simulations of its motion and behavior [35].

**Figure 11:**
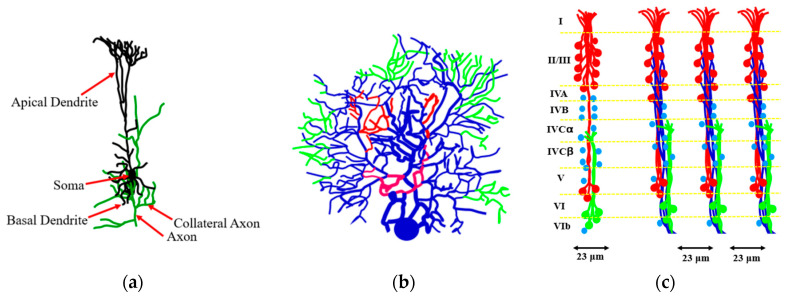
Neurons and their parts forming a network. (**a**) The arrangement of neurons, dendrites, and axons in vertical modules of the striate cortex of the macaque monkey. (**b**) The arrangement of the apical dendrites of pyramidal cells in the cortex showing the six layers (I to VI). The cells in layers II to V (red), VI (green), and (IV) (blue, no dendrites) are shown. (**c**) Columns built of dendrites and axons (based on [36]).

As far as the human brain, the higher-order brain functions, such as cognition, language, sensory perception, and the generation of motor commands are associated with the neocortex. The neocortex is an external part of the brain, which is 3–4 mm thick and with the surface area of 0.26 m^2^. The neocortex is made of six distinct layers of neurons, and it consists of 10^8^ cortical mini-columns with the diameter of about 50–60 μm spanning through all six layers, with about 100 neurons in each mini-column (Figure 11). Although the functionality of the microcolumns (and their very existence) is being debated by some researchers, the columnar structure of the neocortex is widely accepted by most neuroscientists. The microcolumns are combined into the large hyper-columns or macro-columns, which are 300–500 μm in diameter. The hyper-columns have a roughly hexagonal shape, and each column is surrounded by six other columns. Each hyper-column, by some estimates, may include 60–80 microcolumns [36,37,38].

While little is known about the processes inside the microcolumns, it is widely believed that a cortical column can process a number of inputs, and it converts them to a number of outputs using overlapping internal processing chains. Each minicolumn is a complex local network that contains elements for redundancy and plasticity. The minicolumn unites vertical and horizontal cortical components, and its design has evolved specifically in the neocortex. Although minicolumns are often considered highly repetitive clone-like units, they display considerable heterogeneity between cortex areas and sometimes even within a given macrocolumn.

A comprehensive map of neural connections in the brain is called the connectome [39]. A connectome of the *C. elegans* worm has been obtained in 1986, while obtaining a human brain connectome remains a much more challenging task of the scientific discipline referred to as connectomics (compare with genome and genomics or proteome and proteomics). A much more complex connectome of the Drosophila melanogaster fruit fly, a model insect used for various genetic research, whose brain contains about 135,000 neurons and 10^7^ synapses, has been presumably obtained by 2020 [40].

It is believed that the human connectome can be studied at three distinct levels of structural organization: the microscale (connection of single neurons), mesoscale (cortical columns), and the macroscale (anatomical regions of interest in the brain).

Quantitative estimates of the brain network characteristics are remarkable. A measure of the diameter (largest geodesic distance) of the scale-free network of *n* nodes was suggested by Bollobás and Riordan [41] as *D* = log(*n*)/log(log*n*). According to Freeman and Breakspear [42], the neocortical diameter of each hemisphere is close to *D* = 12, for neurons 0.5 × 10^10^ neurons and 10^4^ synapses per neuron yielding *n* = 5 × 10^13^. These numbers are consistent with the idea that at least a three-level hierarchy exists formed by nodes as neurons, hypercolumns, and modules. The reduction of 10^10^ neurons and 10^14^ synapses to a depth of three levels and a diameter of *D* = 12 is viewed as a simplification [42].

Klimm et al. [43] estimated the quantitative characteristics of the human brain network including the hierarchy of the network and its fractal topological dimension. The hierarchy of a network, β, is defined quantitatively by the presumed power law relationship between the node degree and the local clustering coefficient (the ratio of the triangle subgraphs to the number of node triples), $C_{i}\sim k_{i}^{-\beta }$. The fractal dimension, *d*, is a measure of the network’s complexity and is determined by the box-counting method, relating the number of boxes *N_B_* of size *l_B_* that are necessary to cover the network, $N_{B}\sim l_{N}^{-d}$. According to the estimates, the values are β = 0.247 and *d* = 3.7 ± 0.1 [43].

### 6.2. Current Hypotheses on the Formation of the Cortical Network

After discussing the architectural structure of the cortical networks, let us briefly review what is known about the formation of such a complex network, from both an ontogenetic and phylogenetic point of view. The question of to what extent the brain wiring is coded in the DNA remains controversial. The human brain cortex contains at least 10^10^ neurons linked by more than 10^14^ synaptic connections, while the number of base pairs in a human genome is only 0.3 × 10^10^. Therefore, it is impossible that the information about all synaptic connections is contained in the DNA. Currently, two concepts, namely, the Protomap hypothesis and the Radial Unit hypothesis, which complement each other, are employed to explain the formation of the neo-cortex. Both hypotheses were suggested by Pasko Rakic [44].

The Protomap is a term for the original molecular “map” of the mammalian cerebral cortex with its functional areas during early embryonic development when neural stem cells are still the dominant cell type. The Protomap is patterned by a system of signaling centers in the embryo, which provide information to the cells about their position and development. This process is referred to as the “cortical patterning”. Mature functional areas of the cortex, such as the visual, somatosensory, and motor areas are developed through this process.

During the mammalian evolution, the area of the cortical surface has increased by more than 10^3^ times, while its thickness did not change significantly. This is explained by the *Radial Unit* Hypothesis of cerebral cortex development, which was first described by Pasko Rakic [44,45,46]. According to this hypothesis, the cortical expansion is the result of the increasing number of radial columnar units. The increase occurs without a significant change in the number of neurons within each column. The cortex develops as an array of interacting cortical columns or the radial units during embryogenesis. Each unit originates from a transient stem cell layer. The regulatory genes control the timing and ratio of cell divisions. As a result, an expanded cortical plate is created with the enhanced capacity for establishing new patterns of connectivity that are validated through natural selection [36].

An interesting observation about the human connectome was made by Kerepesi et al. [47]. By analyzing the computer data of the “Budapest Reference Connectome”, which contains macroscale connectome data for 418 individuals, they identified common parts of the connectome graphs between different individuals. It was observed that by decreasing the number of individuals possessing the common feature from 418 down to 1, more graph edges appeared. However, these new appearing edges were not random, but rather similar to a growing “shrub”. The authors called the effect the Consensus Connectome Dynamics and hypothesized that this graph growth may copy the temporal development of the connections in the human brain, so that the older connections are present in a greater number of subjects [48].

An important model was suggested recently by Barabási and Barabási [49], who attempted to explain the neuronal connectivity diagram of the *C. elegans* nematode worm by considering neuron pairs that are known to be connected by chemical or electrical synapses. Since synaptic wiring in the *C. elegans* is mostly invariant between individual organisms, it is believed that this wiring is genetically encoded. However, identifying the genes that determine the synaptic connections is a major challenge. Barabási and Barabási [49] identified a small set of transcription factors responsible for the synapses formation of specific types of neurons by studying bicliques in *C. elegans*’ connectome. According to their model, a set of log_2_(*N*) transcription factors is sufficient to encode the connection, if transcription factors are combined with what they called the biological operators.

**Figure 12:**
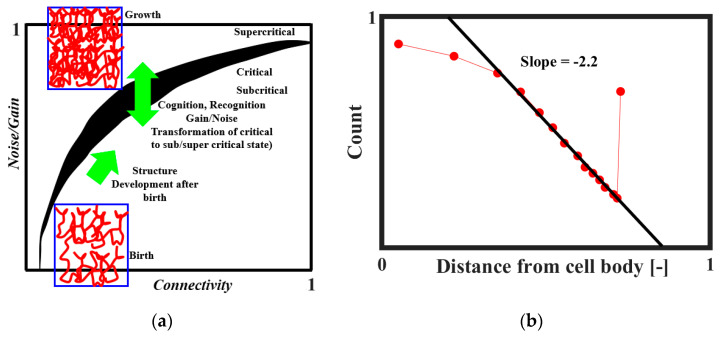
Hypotheses of brain network formation by SOC. (**a**) The neocortex network evolves after the birth toward regions of criticality. Once the critical regions (black) are established, the connectivity structure remains essentially unchanged, but it can adjust close to critical regions (based on [51]). (**b**) Schematic (log-log scale) showing distribution of pyramidal axon tree size. The power law is a typical footprint of scale-free organization [42].

It was proposed that SOC plays a role in the formation of the brain neural network [18,50,51]. The neural connectivity is sparse at the embryonic stage. After the birth, the connectivity increases and ultimately reaches a certain critical level at which the neural activity becomes self-sustaining. The brain tissue as a collective system is at the edge of criticality. Through the combination of structural properties and dynamical factors, such as noise level and input gain, the system may transit between subcritical, critical, and supercritical regimes. This mechanism is illustrated in Figure 12a [51]. The network evolves toward regions of criticality or edge-of-criticality. Once critical regions are established, the connectivity structure remains essentially unchanged. However, by adjusting the noise and/or gain levels, the system can be steered toward or away from critical regions. According to Freeman and Breakspear [42], the power-law distribution of axonal length (Figure 12b) is the evidence of scale-free activity.

Self-organizing critical behavior was reported by Liu et al. [52] for the organization of brain GABA_A_ receptors (these are receptors of γ-Aminobutyric acid or GABA, the major neurotransmitter). The mean size of receptor networks in a synapse followed a power-law distribution as a function of receptor concentration with the exponent 1.87 representing the fractal dimension of receptor networks. The results suggested that receptor networks tend to attract more receptors to grow into larger networks in a manner typical for SOC systems that self-organize near critical states.

### 6.3. Dynamics of Cortical Networks

An amazing feature of brain operations is that they are distributed, rather than localized at a particular neuron or a group of neurons. The distributed operations are performed by a collection of processing units that are spatially separate and communicate by exchanging messages. Mountcastle [36] formulated the following properties of such distributed systems:

- Signals from one location to another may follow any of a number of pathways in the system. This provides the redundancy and resilience.
- Actions may be initiated at various nodal loci within a distributed system rather than at one particular spot.
- Local lesions within a distributed system usually may degrade a function, but not eliminate it completely.
- The nodes are open to both externally induced and internally generated signals.

Various aspects of scaling behavior have been studied for networks associated with the brain, including both special and temporal structures. Several neuroscientists suggested in the 2000s that the human brain network is both scale-free and small-world, although the arguments and evidence for these hypotheses are indirect [42,53], including power-law distributions of anatomical connectivity as well as the statistical properties of state transitions in the brain [54].

Freeman and Breakspear [42] suggested that if neocortical connectivity and dynamics are scale-free, hubs should exist for most cognitive functions, where activity and connections are at a maximum. These hubs organize brain functions at the microscopic and mesoscopic level. They are detectable by macroscopic imaging techniques such as fMRI. Therefore, these are hubs rather than localized functions, which are revealed by these imaging techniques. When connection density increases above a certain threshold, a scale-free network undergoes an avalanche-like abrupt transition and resynchronization almost instantaneously independent of its diameter. Scale-free dynamics can explain how mammalian brains operate on the same time scales despite differences in size ranging to 10^4^ (mouse to whale), as it will be discussed in more detail in consequent sections. Random removals of nodes from scale-free networks have negligible effects; however, lesions of hubs are catastrophic. Examples in humans are coma and Parkinson’s disease from small brain stem lesions [42].

**Figure 13:**
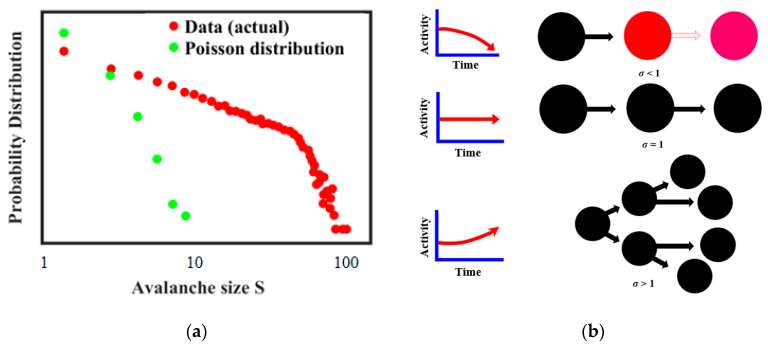
(**a**) Avalanche size (log-log scale) distributions in brain shows a power-law dependency [57] (**b**) The activity may decrease, stay at the same level, or grow with time depending on the branching regime (based on [57]).

Avalanches are a common characteristic of brain signals, along with the so-called bursting [55,56,57]. Neural avalanches show such characteristics as power-law distributions, which is believed to be an indication of near critical behavior [57]. Thus Figure 13a, redrawn from Ref. [57], suggests that the actual size distribution of neuronal avalanches is a power law and it is different from the Poisson model distribution of uncorrelated activities. It is further believed that whether the avalanche occurs depends on the branching regime during the neuron connection (Figure 13b) [57,58,59,60,61].

### 6.4. Default Mode Network and Its Presumed Relations to Cognition

Speaking of the human brain operation, it would be disappointing to avoid such an intriguing topic as what is currently known about the possible connection of brain activities to cognition. While identifying parts of the brain responsible for higher order brain activity, such as cognition or speech, and understanding their mechanisms remains a remote (if at all solvable) task, a number of significant observations have been made in the past 20 years. Some of these observations are related to the temporal scales involved.

In the 1990s, B. Biswal, a graduate student at the Medical College of Wisconsin (MCW), discovered that the human brain displays so-called “resting state connectivity”, which is observable in the fMRI scans [62]. The phenomenon was later called the Default Mode Network (DMN), and it describes brain functions of a resting state. The DMN is active when a person is not focused on the outside world, and the brain is at wakeful rest, such as during daydreaming and mind-wandering. It is also active when individuals are thinking about others, thinking about themselves, remembering the past, and planning for the future. The DMN has been shown to be negatively correlated with other networks in the brain such as attention networks, although the former can be active in certain goal-oriented tasks such as social working memory or autobiographical tasks.

Several recent studies have concentrated upon DMN’s relationship to the perception of a temporal sequence of events, as well as to its role in speech and language-related cognition. These features are of particular interest to the philosophy of mind because language, the ability to plan activities, introspection, and understanding the perspective of another person are often described as distinct characteristic features of humans, which separate them from other mammals.

Konishi et al. [63] investigated the hypothesis that the DMN allows cognition to be shaped by memory-stored information rather than by information in the immediate environment, or, in other words, by “past” rather than by “now”. Using the fMRI technique, they investigated the role of the DMN when people made decisions about where a shape was, rather than where it is now. The study showed that DMN hubs are responsible for the cognition guided by information belonging to the past or to the future, instead of by immediate perceptual input. On the basis of these observations, Konishi et al. [63] suggested that the DMN is employed for higher order mental activities such as imagining the past or future and considering the perspective of another person. These complex introspective activities depend on the capacity for cognition to be shaped by representations that are not present in the current external environment.

In a different study, Lerner et al. [64] investigated how human activities involving the integration of information on various time-scales is related to the DMN activation. During real-time lasting activities, such as watching a movie or engaging in conversation, the brain integrates information over multiple time scales. The temporal receptive window (the length of time before a response during which sensory information may affect that response) becomes larger when moving from low-level sensory to high-level perceptual and cognitive areas. Lerner et al. [64] showed that the temporal receptive window has a hierarchical organization with levels of response to the momentary input, to the information at the sentence time scale, and to the intact paragraphs that were heard in a meaningful sequence. The researchers further hypothesized that the processing time scale is a functional property that may provide a general organizing principle for the human cerebral cortex.

In a neurolinguistics study, Honey et al. [65] performed fMRI research to figure out whether different languages affect different patterns in neural response. They made bilingual listeners hear the same story in two different languages (English and Russian). The story evoked similar brain responses, which were invariant to the structural changes across languages. This demonstrated that the human brain processes real-life information in a manner that is largely insensitive to the language in which that information is conveyed. Simony et al. [66] further investigated how the DMN reconfigures to encode information about the changing environment by conducting fMRI while making subjects listen to a real-life auditory narrative and to its temporally scrambled versions. The momentary configurations of DMN predicted the memory of narrative segments. These are interesting studies that may provide insights into questions such as how the natural language is related to the hypothetical language of thought, which has been postulated by some cognitive scientists and philosophers of language.

### 6.5. Information and Entropic Content of the Network

The suggestion that the brain’s connectome as a network possesses topological properties of fractal, scale-free, or small-world networks brings a number of interesting questions. The hierarchically organized network with the fractal dimension of *d* (some estimates state the value of the fractal dimension *d* = 3.7 ± 0.1 [43]*)* is packed into the 3D cortex, forming a thin layer whose thickness is almost two orders of magnitude smaller than its two other dimensions.

Barabási and Barabási [49] noted that in order to store the information about the exact structure of the connectome of *N* neurons, a neuron with *k* links would need *k*·log_2_(*N*) bits of information, with the total information in all neurons *kN*·log_2_(*N*). For large organisms, this would significantly exceed the information contained in DNA (Table 3). Thus, 3.1·10^21^ bits of information would be required to characterize the human brain; for comparison, some estimates indicate that human brain memory capacity is 10^15^–10^16^ bit, while the human genome has about 10^11^ pairs of nucleotides.

To overcome this difficulty, Barabási and Barabási [49] suggested a selective coding model. According to their model, the brain cannot encode each link at the genetic level. Instead, selective operators are employed, which inspect the transcription factor signatures of two neurons (somewhat close in space) and facilitate or block the formation of a directed link between them. This can be achieved by external agents, such as glia cells, which select specific neurons and facilitate synapse formation between them or detect the combinatorial expression of surface proteins, whose protein–protein interactions catalyze synapse formation. The action of such selective operators is evidenced by the emergence of detectable network motifs in the connectome, namely, bicliques.

The model by Barabási and Barabási [49] could provide an insight into the question of how much of the information in the structure of the brain is contained in the DNA and how much is generated during the embryonal and post-embryonal development by self-organizing processes. The lower boundary of genetic information can be estimated by the number of transcription factors, *T*, which encode the identity of a neuron times the total number of neurons.

## 7. Time Scale of Neuronal Activities

In this section, we will review current experimental data about the scaling properties of cortical networks related to their spatial and temporal organization and their informational content from the entropic viewpoint. There are several approaches to what constitutes a “time constant” for the brain and how this time constant (i.e., the rate of neural processes) scales with the size of an animal.

### 7.1. The Critical Flicker Fusion Thresholds

Brain networks show a number of remarkable properties. One important experimental observation is that despite the difference in their size by 10^4^ times from a mouse to a whale, mammalian brains tend to operate at almost the same time scales. This can be called the law of conservation of the characteristic time scale. There are two approaches to the characterization of the time scale of brain activity of different creatures: studying brain waves (rhythms of oscillation) and investigations of the critical flicker fusion (CFF) thresholds. The CFF is defined as the frequency of an intermittent light, at which the light appears steady to a human or animal observer (similar to frames in the cinema). It has been hypothesized that the ability of an animal to change their body position or orientation (manoeuvrability) is related to the ability to resolve temporal features of the environment and eventually to the CFF [67]. Manoeuvrability usually decreases with increasing body mass.

Buzsáki et al. [68] reviewed the temporal scaling properties of cortical networks by studying the hierarchical organization of brain rhythms in mammals. The brain is known to generate electromagnetic oscillations of different frequencies, which can be observed with the EEG. While the exact nature and function of these oscillations remains debatable, they are highly reproducible and classified by their frequencies: alpha waves (7–15 Hz), beta waves (15–30 Hz), gamma waves (>30 Hz), and others. The frequency of brain oscillations covers almost five orders of magnitude, from <0.01 Hz to hundreds of Hz. The power distribution of brain oscillations tends to show the 1/*f^n^* (where *f* is the frequency and *n* is the power exponent) noise spectrum when measured at long-scale ranges. As we have discussed in the preceding sections, such a distribution spectrum is a signature of SOC. However, when specific brain activities are considered, such as concentrating on particular features, moving or orienting in space or various cognitive functions, particular oscillation frequencies become dominant, and the spectrum deviates from the 1/*f* or 1/*f^n^* statistics, showing peaks at some characteristic frequencies. These frequencies of various rhythm classes do not vary significantly with the size of the brain [68].

As far as modeling the origin of oscillations, several dynamic models have been employed to study brain rhythms at various scales and, in particular, at the mesoscale. Thus, the so-called Freeman’s *K*-sets model follows the Katzir–Katchalsky suggested treatment of cell assemblies using network thermodynamics. Hierarchical models of several layers of these sets, from *K*-I to *K*-V, provide a modeling tool to conduct analyses of the unifying actions of neocortex related to intentional and cognitive behaviors [42].

The correlation of spatial and temporal brain activity organization was studied by Honey et al. [65]. They related the spontaneous cortical dynamics to the underlying anatomical connectivity at multiple temporal scales. Structural (spatial) hubs corresponded to the hubs of the long-run (minutes) neural activity. For the activities with shorter characteristic time (seconds), significant deviations from special hubs were observed. At the shorter time scale (fraction of a second), individual episodes of interregional phase-locking were detected.

**Figure 14:**
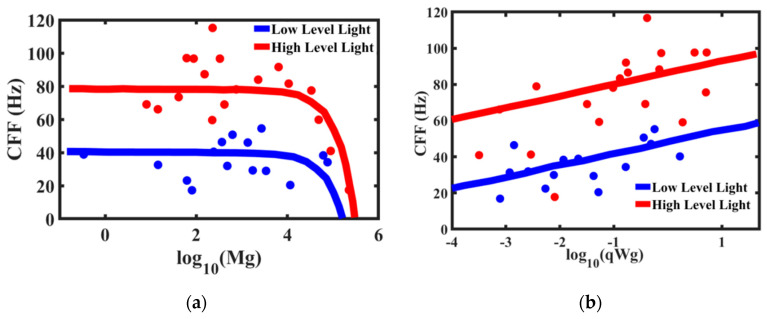
The effect of (**a**) body mass (gram) and (**b**) temperature-corrected mass-specific resting metabolic rate (qWg) on the critical flicker fusion (CFF) shows that the CFF increases with the metabolic rate but decreases with body mass (based on [67]).

The critical flicker fusion threshold is viewed by many researchers as the time scale (or frequency) at which the brain operates. The higher frequency of the CFF threshold implies the brain’s ability to discern signals and react faster. Healy et al. [67] studied the effect of body mass and metabolic rates of various species on their CFF threshold (Figure 14). The comparative metabolic rates are determined separately by measuring oxygen consumption through ventilation [69]. It is expected that smaller animals have higher temporal resolution. Larger animals respond to a stimulus slower than the smaller animals. Therefore, high temporal resolution in larger animals is unnecessary. On the other hand, faster and more manoeuvrable fly species have higher temporal resolutions [70], which makes it, for example, so difficult for a human to catch a fly. Note that the mass-specific metabolic rates are almost constant (or, more accurate to say, lie within a certain relatively narrow range) for different life forms with 20 orders of magnitude difference in the body mass [68]. Furthermore, the accuracy of the 3/4-power allometric scaling Kleiber law for metabolic rate is not considered universal by some scholars [4].

We conclude that the characteristic frequencies of brain activity are either almost constant or slightly decrease with increasing body and brain size.

### 7.2. Brain Rhythms and Scaling

The allometric approach can be applied to the inter-species analysis of brain activity frequencies and time scales in both humans and various species. The observation that typical frequencies of brain activities are often independent of brain size requires an explanation. When brains of various species are compared, the distance between homologous regions of the brain increases with the growing size of the brain. Moreover, the number and length of axons increase even more rapidly than the number of neurons with the growing brain size. Consequently, the average number of synaptic connections in the shortest path between two neurons (the “synaptic path length”) also grows in a very fast manner. Assuming that the cortical network is a scale-free and/or a small-world network would decrease the path length; however, it does not eliminate completely the scale dependency. The mechanisms that facilitate the increase of signal speed in larger species should be investigated.

Such an investigation was suggested by Buzsáki et al. [68]. They used experimental data about the scaling of the brain, in particular, those presented by Wang et al. [71] on the size of axons and the amount of white matter in the brain. These are parameters that affect the signal speed. The experimental observations indicate that the increase in axon caliber (size) and their insulation (myelination) compensates for the increased time of signal transfer. The volume of the myelin or white matter in relation to the gray matter (neurons) in the brain scales as power 4/3 with the size. For instance, while the white matter constitutes 6% of the neocortical volume in hedgehogs, in humans, it exceeds 40% of brain volume. This is because a larger brain requires faster signal propagation, and the degree of myelination in larger brains increases.

The velocity of propagation of the neural signal is another significant parameter. Buzsáki et al. [68] noted that for the phase synchronization of gamma waves in a mouse (brain size approximately 5–10 mm), the speed of conduction of 5 m/s is sufficient, while for humans (70–140 mm), much larger conduction speeds are needed [60]. While an increase in conduction velocity can be achieved by both the increase of the volume fraction of white matter and of the axon size, there are several problems associated with this approach.

**Figure 15:**
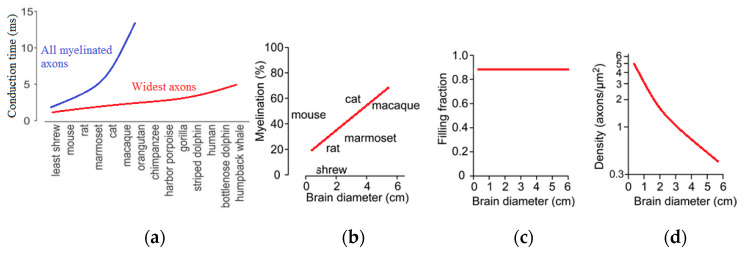
Interspecies scaling relations in the brain (based on [68] and [71]). (**a**) Cross-brain conduction times for myelinated axons; (**b**) the fraction of myelinated axons; (**c**) the fraction of volume filled by axons; (**d**) distribution of axon densities.

An increase of axon diameter proportional to the size of the brain would enormously increase the brain volume. Experimental observation suggests that, apparently, only a small fraction of all axons have large diameters. Particularly, the largest axons scale linearly with the brain size (Figure 15), and they result in an increase of the connection speed [68,71]. At the same time, the required increase of the white matter volume in larger brains does not lead to an unreasonable growth of the volume, because only a small fraction of all axons is large. Consequently, despite a 17,000-fold difference in mammalian brain volumes, the oscillation rhythms with their typical time scales independent of the brain size are supported by the same mechanisms, and they still have the same typical frequencies.

The number of nodes, *N*, scales proportionally to the power *D* of the characteristic length size of the brain, *l*, as

(6) $$N\;\sim \;l^{D}$$

where *D* is the fractal dimension of the network. The velocity of neural signal propagation is dependent on the ratio *w* = *W*/*G* of the volume fractions of the white matter, *W*, to the gray matter, *G*, as a power function,

(7) $$V\;\sim \;w^{d}\;\sim \;{\left(\frac{W}{G}\right)}^{d}.$$

As we have discussed in the preceding sections, in small-world networks, the distance between two random nodes is proportional to the logarithm of the total number of nodes,

(8) L ~ln(N)~ D·ln(l). 

The rate of neural processes, i.e., the time scale of the brain activity is related to the size of an animal. However, given the experimental observation that the rate is almost a constant independent of the size of the brain, one can assume that the volume fraction of white-to-gray matter affects the velocity of the signal

(9) $$\tau \;\sim \;\frac{L}{V}\;\sim \;\frac{D\cdot \ln\left(l\right)}{{\left(\frac{W}{G}\right)}^{d}}=\mathrm{const}.$$

From Equation (9), it follows immediately that

(10) $$\frac{D\cdot \ln\left(l\right)}{{\left(\frac{W}{G}\right)}^{d}}=\mathrm{const}$$

which can be presented as

(11) $$w=\frac{W}{G}=D^{\frac{1}{d}}*{\left(\ln\left(l\right)\right)}^{\frac{1}{d}}\;.$$

**Figure 16:**
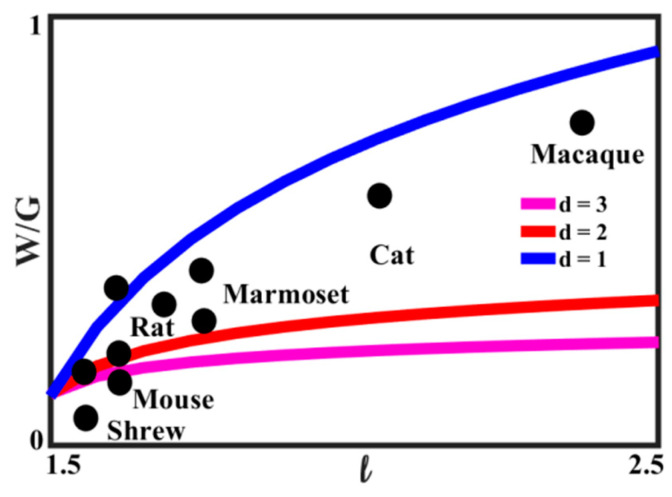
Scaling relationship between the brain diameter (cm) and the ratio of white and gray matter.

Equation (10) relates the ratio of white-to-gray matter to the characteristic size of the brain. The relationship is plotted in Figure 16 for the value of *D* = 3.2, for several values of the exponent *d*. The experimental data, based on [71], for several animals are also presented. It is seen that the best agreement with Equation (11) is for 2 < *d* < 3, which is consistent with the concept that the growth of the white matter content compensates for the increasing linear size of the brain.

## 8. Immune Networks and Artificial Neural Networks

An interesting spin-off of the network approach to the neural science has been developed in the field of immunology, where Niels Jerne [72] and Geoffrey Hoffmann [73] have suggested the so-called Immune Network Theory (INT). According to this theory, the immune system of a human or of an animal is a network of lymphocytes (immune cells) and molecules (antibodies, such as immunoglobulins), which interact with each other. An invasion of a foreign antigen *A* (which may be a virus, microbe, protein, or even an inorganic compound) activates immune cells and molecules *anti-A*, which, in turn, activate *anti-anti-A*, and so on. The nodes of the network are immune cells, antibodies, and antigens, while the edges are interactions between them. Therefore, the reaction of the immune system on the antigen is somewhat similar to the reaction of a network upon a stimulus applied to one of its nodes: it may be a local perturbation or an avalanche-type response affecting many nodes of the system.

According to Jerne, there are many similarities between the immune system and the central nervous system. The numbers of cells in both systems are of the same order, 10^10^–10^11^. The mass of immune cells in a human is on the order of 1 kg, which is somewhat comparable with the brain mass, at least, by the order of magnitude. Both the immune and nervous systems respond to external stimuli, and they both possess memory [73].

The INT theory was suggested in the 1970s. Although Jerne became a winner of the 1984 Nobel Prize for his work in immunology, the INT remains a hypothesis that may require further experimental validation. Jerne apparently sought an analogy of the INT not only with the principals of cortical network organization, but also with the rules, which govern human language, such as the Chomskian concept of the generative grammar. Jerne’s Nobel Lecture is entitled “The Generative Grammar of the Immune System” [72].

Since the turn of the 21st century, there have been attempts to investigate the scaling properties of the immune network, including their scale-free and fractal properties [74,75]. This is often conducted in the context of a broader area of the protein networks, since the interactions between antigens and antibodies or immune cells are bio-specific (ligand–receptor) protein–protein interactions [76]. Conceptually, these efforts are supposed to give insights, for example, on autoimmune diseases. One can hypothesize that, similarly to the SOC sand-pile model, where the addition of a sand grain usually has a local effect, but sometimes it can cause avalanches, the reaction of the immune network to an external stimulus (a pathogenic antigen) can be an immune response or a catastrophic series of events leading to a disease. However, the INT still largely misses a connection with experimental science. The term “immunome” (and therefore, the area of “immunomics”) has already been suggested [77] as an analogy for the genome (and genomics), proteome (and proteomics), and connectome (and connectomics).

As far as the protein networks, it has been shown that hydrophobic interactions during protein folding result in the SOC mechanisms that can explain scaling properties and power-law exponents (e.g., size–volume dependencies) in proteomics [78]. The relation of hydrophobic interactions and SOC has been established [14], and similar considerations have been used in materials science, in particular, for the design of anti-icing coatings [79].

Networks of cortical neurons in the brain are a source of inspiration for the area of biomimetic ANN. Computational algorithms inspired by the INT have been suggested as well [80]. According to the clonal selection theory of immunity, when a pathogen invades the organism, immune cells (lymphocytes) that recognize these pathogens would proliferate yielding effector cells with secrete antibodies (immunoglobulin proteins) and memory cells. The multitude of available immune cells is explained as a result of somatic mutations with high rates, while pathogens drive their selection force. A rough model of this process is used as a basis for genetic computational algorithms called the Artificial Immune Systems (AIS) algorithms [81].

## 9. Conclusions

Methods of the network science and information theory can be used for the analysis of diverse types of physicochemical and biological systems. The systems reviewed in this article include granular materials, droplet clusters, colloidal crystals, artificial neural networks, biological vascular networks, cortical networks of neurons connected by synapses, and immune networks. Scaling, topology, and dimensional analysis can apply to networks that describe these different physical and biophysical systems. Some of these networks possess fractal, scale-free, and small-world scaling properties. Others exhibit different types of scaling relationships often involving power laws. The amount of information contained in a network can be found by calculating its Shannon entropy.

We discussed the properties of colloidal networks arising from small granular and colloidal particles and from droplet clusters due to pairwise interaction between the particles. Many networks found in colloidal science possess self-organizing properties due to the effect of percolation. The force networks in packed granular material leading to the jamming transition are a typical example. Other systems may exhibit self-organized criticality. They are brought to a critical point by a combination of slow motion and a dissipation mechanism, which can balance out. These systems have critical states, which have distinct signatures of fractal dimensions and power laws (often one-over-frequency spectrum), although, of course, not every system with a one-over-frequency spectrum is an SOC system. Colloidal systems exhibit various scaling relationships including the fractal (scale-free), Zipf, Lewis, Desch, and Aboav scaling laws.

Branching vascular systems demonstrate the allometric power exponents of ¾. This power exponent in such systems is explained by a fractal model of branching with the simultaneous conservation of the volume served by blood vessels at different levels and the flow rate.

Then, we discussed much more complex networks of neurons, which are organized in the neocortex in a hierarchical manner, forming micro- and macro-columns. The scaling relationships in these networks suggest that the characteristic time constant is independent of brain size when interspecies comparison is conducted. This is because the increased diameter of the network is compensated by the increasing velocity of the signal due to myelination (the insulation of neurons by the white matter). The characteristic time constant can be defined in terms of the frequency of different types of brain waves or as the CFF threshold.

The brain networks possess many characteristics typical to other networks, including the one-over-frequency and power-law activities, avalanches, small-world, scale-free, and fractal topography. It is particularly interesting to look for the correlation between the spatial distribution (for example, hubs) and temporal organization (frequency spectrum) of human brain cognitive activities. Such research is being conducted by many groups, for example, the study of the DMN during such activities as the comprehension of a text in a natural language versus contemplating it (the “language of thought”).

The information content of the neural networks can be studied using the standard characteristics of the information theory, such as the Shannon entropy. It may provide ways to distinguish between DNA-encoded information and information generated during the embryonal and post-embryonal development, which may be driven by the self-organizing process.

For engineers and computer scientists, neural networks serve as a source of inspiration for artificial neural networks, which can serve as a means of machine learning and establishing correlations in data-reach areas, such as surface engineering. Cortical networks also served as a source of inspiration for the concept of the immune network, which, in turn, became an inspiration for Artificial Immune Systems algorithms in computer science. 

Both network science and brain physiology are dynamic, rapidly expanding fields. New approaches are likely to emerge. For example, in the study of small droplet clusters, unusual properties, such as the applicability of the Dynkin diagrams for colloidal studies have been suggested. A huge amount of information has been obtained in recent studies about both the structure and properties of biological and artificial networks. There are still many questions and problems remaining, such as obtaining connectomes of various species, understanding the relation between the genomic information and organization of cortical networks, and the internal organization of the latter.

## Figures and Tables

**Table 1:** Structures of eight-bond colloidal clusters and their magnitudes of probability distribution and Zipf-Law distribution (data from [28]).

	A	B	C	D	E	F	G	H
Structures	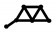	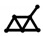	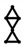	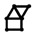	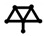	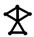	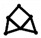	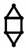
Probability	0.35 ± 0.1	0.23 ± 0.06	0.11 ± 0.05	0.10 ± 0.02	0.07 ± 0.01	0.08 ± 0.02	0.07 ± 0.01	0.05 ± 0.0
Zipf-Law	0.36	0.19	0.14	0.10	0.08	0.07	0.06	0.05

**Table 2:** Structures of seven-bond colloidal clusters and their magnitudes of probability distribution and Zipf-Law distribution (data from [28]).

	**A**	**B**	**C**	**D**	**E**	**F**	**G**	**H**
Structure	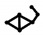	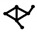	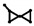	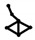	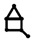	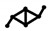	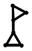	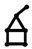
Probability	0.20 ± 0.05	0.17 ± 0.03	0.18 ± 0.03	0.09 ± 0.03	0.07 ± 0.01	0.05 ± 0.01	0.05 ± 0.02	0.03 ± 0.001
Zipf Law	0.21	0.16	0.14	0.10	0.09	0.07	0.05	0.04
	**I**	**J**	**K**	**L**	**M**	**N**	**O**	**P**
Structures	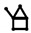	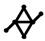	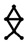	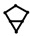	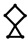	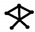	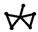	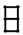
Probability	0.04 ± 0.002	0.025 ± 0.002	0.045 ± 0.002	0.026 ± 0.002	0.015 ± 0.001	0.005 ± 0.0	0.005 ± 0.0	0.01 ± 0.001
Zipf Law	0.03	0.02	0.02	0.02	0.01	0.007	0.007	0.01

**Table 3:** Number of neurons, synapses, and corresponding information content of some organisms (based on [49]).

Organism	Neurons, *N*	Synapses, *k*	Bits Per Neuron, *k*·log_2_(*N*)	Bits Per Connectome, *kN*·log_2_(*N*)	Transcription Factors, *T*	Transcription Factors Information, *N·T*
*C. elegans*	302	6398	3818	1.3·10^6^	934	2.8·10^5^
*Drosophila* fruit fly	10^5^	10^7^	2.3·10^6^	2.3·10^11^	627	6.3·10^7^
Mouse	7.1·10^6^	1.3·10^11^	2.6·10^8^	1.9·10^15^	1457	1.0·10^10^
Cat	7.6·10^8^	6.1·10^12^	3.2·10^10^	2.5·10^19^	887	6.7·10^11^
Human	8.1·10^9^	1.6·10^14^	3.8·10^11^	3.1·10^21^	1391	1.1·10^13^
